# Phytochemical Analysis and Antioxidant Activities of Various Extracts from the Aerial Part of *Anemone baicalensis* Turcz.: In Vitro and In Vivo Studies

**DOI:** 10.3390/molecules29194602

**Published:** 2024-09-27

**Authors:** Shuang Sun, Guangqing Xia, Hao Pang, Junyi Zhu, Li Li, Hao Zang

**Affiliations:** 1 College of Pharmacy, Yanbian University, Yanji 133000, China; ss11721@163.com (S.S.); qingguangx@thnu.edu.cn (G.X.); 2 School of Pharmacy and Medicine, Tonghua Normal University, Tonghua 134002, China; panghao0447@163.com (H.P.); swx0527@163.com (J.Z.); 3 Key Laboratory of Evaluation and Application of Changbai Mountain Biological Gerplasm Resources of Jilin Province, Tonghua 134002, China; 4 School of Pharmaceutical Engineering, Shenyang Pharmaceutical University, Benxi 117004, China

**Keywords:** *Anemone baicalensis* Turcz., chemical composition, antioxidant properties, gastroprotective effects

## Abstract

*Anemone baicalensis* Turcz., a botanical species with a rich historical background in traditional medicine for detoxification and insecticidal applications, possesses a vast, yet largely unexplored, therapeutic potential. This study primarily focused on conducting a qualitative phytochemical analysis of the plant, determining the active ingredient content and antioxidant activity of various solvent extracts. The qualitative phytochemical analysis revealed the presence of 12 different types of phytochemicals within the plant. Utilizing ultraviolet-visible spectrophotometry, we identified 11 active ingredients in 4 solvent extracts. Notably, the methanol extract was found to contain high concentrations of total carbohydrate, total monoterpenoid, total phenolic, total tannin, and total triterpenoid. In the antioxidant experiment, the methanol extract demonstrated superior scavenging abilities against 1,1-diphenyl-2-picrylhydrazyl radical, 2,2′-azino-*bis*(3-ethylbenzothiazoline-6-sulfonicacid) diammonium salt, superoxide anion radical, and hydrogen peroxide, outperforming other extracts in chelation experiments aimed at reducing iron and metal ions. Consequently, the methanol extract was selected for further investigation. Subsequent ultrahigh-performance liquid chromatography-electrospray ionization-quadrupole-time of flight-mass spectrometry analysis revealed that the methanol extract contained 39 compounds, primarily phenolic compounds and triterpenoid saponins. Three stability assessments confirmed the extract’s stability under high temperatures, varying pH levels, and simulated gastrointestinal processes. Additionally, oil stability testing demonstrated its antioxidant capacity in extra virgin olive oil and cold-pressed sunflower seed oil media. An oral acute toxicity experiment conducted on mice not only confirmed the absence of acute toxicity in the methanol extract but also provided a dose reference for subsequent gastric protection experiments. Notably, the methanol extract exhibited significant gastroprotective effects against ethanol-induced gastric lesions in rats, as evidenced by histopathological and biochemical analyses. Specifically, the extract reduced levels of malondialdehyde, alanine aminotransferase, and aspartate aminotransferase while increasing glutathione, nitric oxide, and catalase, indicating its gastroprotective mechanism. These findings suggest that the methanol extract from the aerial part of *Anemone baicalensis* could be a promising therapeutic agent for conditions associated with oxidative imbalances. They underscore the plant’s potential therapeutic benefits and offer valuable insights into its antioxidant properties, thereby broadening our understanding of its medicinal potential.

## 1. Introduction

The genus *Anemone* L. encompasses over 150 species globally, with approximately 52 of these species residing in China. Predominantly found in the underbrush of mountainous forests and shrubbery in the northeastern and southwestern regions of China [1]. *Anemone* plants have garnered significant research attention in recent years. A review has summarized the presence of various beneficial compounds, including triterpenoid saponins, organic acids, and coumarins, with triterpenoid saponins being identified as the primary active constituents [2]. These compounds possess potent anti-tumor, antioxidant, and anti-bacterial properties [3], positioning *Anemone* plants as a promising source of triterpenoid saponins. Consequently, there has been a heightened interest in investigating triterpenoid saponins within this genus to further expand their medicinal potential. Notably, diverse triterpenoid saponins have been isolated from specific *Anemone* species, such as *Anemone begoniifolia* [4], *Anemone rivularis* [5], *Anemone cathayensis* [6,7], *Anemone taipaiensis* [8,9], and *Anemone hupehensis* [10], either from their aerial part or the whole plant. Furthermore, advanced research employing high-performance liquid chromatography (HPLC) methods has identified the triterpenoid saponin raddeanoside R3 in various parts of *Anemone raddeana*, revealing comparable concentrations in both leaves and rhizomes [11]. As research continues to evolve, it has become increasingly apparent that *Anemone* plants exhibit a wide range of pharmacological activities, including anti-inflammatory, analgesic, antioxidant, anti-tumor [7,12,13,14], and anti-bacterial effects [7,13,15,16]. These discoveries highlight the significant research potential residing in the aerial part of *Anemone* plants, emphasizing the importance of continued exploration in this field.

**Figure 1:**
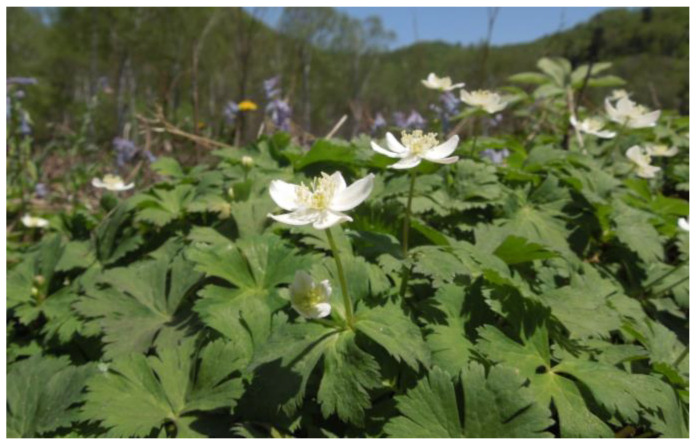
Morphology of *Anemone baicalensis* aerial part.

*Anemone baicalensis* Turcz. (*A. baicalensis*) (Figure 1), typically attains a height of 13–28 cm and blossoms from May to July amidst the undergrowth of mountainous forests, shrubbery, or wetlands located on shady slopes. Its distribution extends across altitudes ranging from 500 to 900 m in Chinese provinces, including Jilin, Heilongjiang, Liaoning, Sichuan, Shaanxi, and Yunnan, as well as in eastern Siberia and Russia [1,17]. Conventionally, the whole plant is gathered during the summer months, meticulously cleaned, and subsequently dried for future use. It is renowned for its detoxifying and insecticidal properties [18], prompting us to investigate its potential toxicity and use it responsibly in accordance with safety guidelines in the future. Despite the abundance of wild resources available, there is a notable scarcity of literature detailing the chemical composition and biological activities of *A. baicalensis*, thereby presenting a compelling opportunity for further exploration. The current study endeavors to thoroughly investigate the *A. baicalensis* aerial part (ABAP), with a primary emphasis on elucidating its chemical composition and assessing its antioxidant potential both in vitro and in vivo. This research offers significant evidence for the potential application of *A. baicalensis* in the treatment of oxidative stress-related diseases.

## 2. Results and Discussion

### 2.1. Preliminary Identification of Phytochemicals

In this study, a thorough qualitative phytochemical analysis was undertaken on ABAP, as presented in Table S1. The outcomes of this analysis revealed that anthraquinones, cardiac glycosides, and cyanogenic glycosides were absent from the plant material. As for the evaluation of flavonoids, tannins, alkaloids, coumarins, and lactones, our research encountered mixed results, with certain experiments yielding positive findings and a few others yielding negative outcomes. These mixed results are intriguing and suggest that, while these four phytochemicals may indeed be present in the plant, their concentrations are likely to be low. Given the inherent variability in the sensitivity of individual experimental methods, it is plausible that those with lower sensitivity thresholds might have failed to detect their presence, yielding negative results. Nonetheless, this hypothesis necessitates rigorous validation through subsequent quantitative phytochemical analysis to ascertain the precise concentrations of these compounds in ABAP.

### 2.2. Extraction Yields of Different Extracts of ABAP

**Figure 2:**
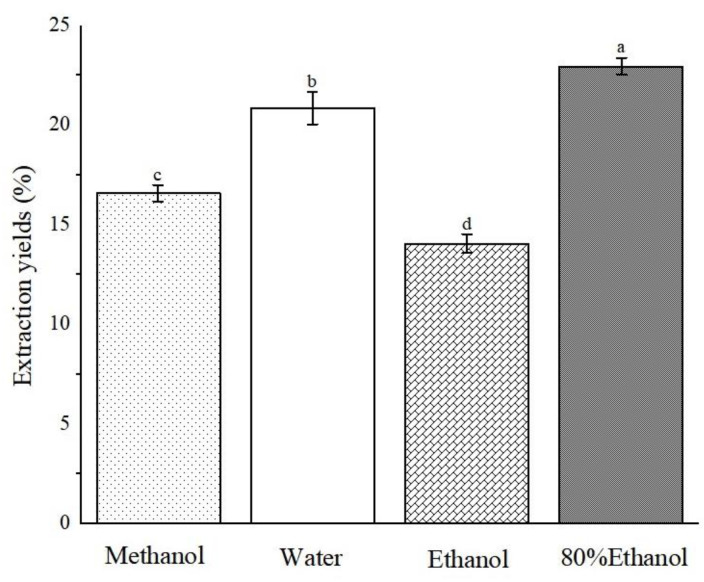
Extraction yields of *Anemone baicalensis* aerial part extracted with four solvents. ^a–d^ Columns with different superscripts indicate a significant difference (*p* < 0.05).

Through examination of various plant species, we discerned a discernible pattern as follows: polar solvent extracts frequently exhibited antioxidant activity [19,20,21]. Therefore, in this study, ABAP were extracted using a range of solvents, namely, methanol, water, ethanol, and 80% ethanol, with the objective of analyzing the content of various active ingredients and antioxidant properties present in the four solvent extracts. Following the completion of the extraction process, we employed vacuum distillation to thoroughly evaporate the residual solvent. Rigorous protocols were implemented in subsequent steps to guarantee the complete elimination of solvent residues, thereby mitigating any adverse effects on our experimental outcomes or their potential applications. As evident from Figure 2, the 80% ethanol extract achieved the highest extraction rate, reaching 22.96% ± 0.43, closely trailed by aqueous and methanol extracts. In contrast, the ethanol extract displayed the lowest extraction rate, at a mere 14.06% ± 0.47. Notably, there is a substantial variation in the yield of extracts obtained using different solvents. It is noteworthy that solvents containing water exhibited a higher extraction rate, which can likely be attributed to the high polarity of water, enabling it to dissolve impurities such as pigments and pectin more efficiently. This observation emphasizes the crucial role of solvent selection in enhancing the efficiency of extracting bioactive compounds from plant materials.

### 2.3. Content of Active Ingredients

#### 2.3.1. Total Carbohydrate Content (TCC)

Carbohydrates are essential constituents of biological cells, functioning as their primary source of energy [22]. Furthermore, they play a pivotal role in enhancing gastrointestinal motility and alleviating the detrimental effects of bacteria and toxins on the intestinal tract [23]. Among carbohydrates, glycosides display a broad range of pharmacological properties. For instance, raddeanoside R3, derived from *Anemone raddeana*, exhibits anti-tumor, anti-inflammatory, and analgesic activities [11]. The present study evaluated the carbohydrate content in various solvent extracts of ABAP and discovered that the TCC ranged from 355.38 ± 2.86 to 433.95 ± 5.17 mg glucose equivalents (GE)/g extract, as summarized in Table 1. Significant differences were noted among the groups, with the ethanol extract yielding the highest TCC, followed by the methanol extract, 80% ethanol extract, and aqueous extract. Notably, the TCC in extracts containing water, such as the aqueous extract and 80% ethanol extract, was lower, potentially due to the plant’s lower carbohydrate content and correspondingly higher glycoside content. The polarity of water significantly influences the efficiency of glycoside extraction. The carbohydrate content data indicates that the water extract contains the least amount of carbohydrates. But water, as a solvent, excels at extracting highly polar carbohydrates like polysaccharides and oligosaccharides. Conversely, glycoside compounds, such as triterpenoid saponins, with their relatively low polarity, are not readily soluble in water. In contrast, organic solvents like ethanol or methanol, with their superior solubility properties, can more efficiently extract a diverse array of sugar-related compounds, including glycosides.

#### 2.3.2. Total Protein Content (TP_ro_C)

Plant protein is a highly valuable nutrient for human health, offering numerous unique health benefits. In contrast, animal protein often contains elevated levels of saturated fat and cholesterol, and excessive intake can exacerbate hypercholesterolemia [24]. However, plant protein successfully avoids this issue associated with animal protein. Prolonged consumption of plant protein has been shown to enhance physical fitness and contribute positively to overall wellbeing. Additionally, it has been proven to lower blood lipids and improve cardiovascular health [25]. Our current study revealed that the aqueous extract of ABAP possessed the highest TP_ro_C, reaching 493.08 ± 3.15 mg bovine serum albumin equivalents (BSAEs)/g extract (Table 1). This finding highlights the substantial presence of plant protein in ABAP, presenting exciting opportunities for research and development of health-promoting products.

#### 2.3.3. Total Monoterpenoid Content (TMC)

Monoterpenoids, a class of compounds predominantly found in diverse volatile oils, exhibit a myriad of pharmacological effects, including anti-bacterial, antiviral, and antioxidant properties [26,27]. In this study, the TMC of various ABAP extracts was determined and found to be exceptionally high, ranging from 1152.54 ± 19.05 to 1622.28 ± 15.72 mg linalool equivalents (LE)/g extract (Table 1). This remarkable revelation emphasizes the abundance of monoterpenoids in ABAP and their potential for therapeutic applications. Furthermore, qualitative phytochemical analysis utilizing the CH_3_COOH-CuSO_4_ test confirmed that the monoterpenoids belong to the unique class of iridoid glycosides, a specialized subclass of monoterpenoids that have garnered considerable attention due to their diverse therapeutic applications in treating conditions such as depression, anxiety, tumors, and diabetes [28]. This revelation not only enhances our knowledge of the chemical makeup of ABAP but also opens up new possibilities for innovative research directions. Given the encouraging therapeutic potential of iridoid glycosides, future studies should strive to undertake a comprehensive examination of the volatile components of *A. baicalensis*, with a particular emphasis on identifying and characterizing the specific iridoid glycosides present. Additionally, evaluating the efficacy of these compounds in managing depression and anxiety, along with elucidating their mechanisms of action, would provide invaluable insights into their therapeutic potential.

#### 2.3.4. Total Alkaloid Content (TAC)

Alkaloids, a class of nitrogen-containing organic compounds ubiquitous in plants, are renowned for their potent anti-tumor and anti-inflammatory effects [29,30]. In this study, the TAC of four distinct extracts was quantitatively assessed using spectrophotometry, revealing a substantial variation in concentrations, ranging from 3.00 ± 0.00 to 5.71 ± 0.01 mg berberine hydrochloride equivalents (BHE)/g extract (Table 1). Notably, the ethanol extract exhibited the highest TAC, closely followed by the methanol extract, aqueous extract, and 80% ethanol extract, respectively. Previous studies have emphasized the ability of alkaloids to reduce the production of reactive oxygen species (ROS), inhibit the activity of nitric oxide synthase 2, and exhibit robust antioxidant activity [31]. Our quantitative analysis validates the presence of alkaloids in all tested extracts, suggesting that the lack of detection in qualitative analysis may be attributed to sensitivity limitations. Moreover, the results indicate that although alkaloids are present in this plant, their content is relatively low and does not constitute the primary component. This finding emphasizes the minimal safety risk posed by the low alkaloid content. Additionally, preserving these low levels does not detract from the plant’s primary uses or functionality, thereby ensuring its ongoing efficacy and safe application.

#### 2.3.5. Total Phenolic Content (TP_he_C)

Phenolic compounds, secondary metabolites abundant in the sun-exposed aerial parts of plants like stems, fruits, leaves, and flowers, are renowned for their myriad health benefits to humans [32]. These benefits encompass potent antioxidant, lipid-lowering, and immune-enhancing effects [33]. In this study, the TP_he_C of four solvent extracts of ABAP was quantitatively assessed (Table 1). The TP_he_C values varied significantly, ranging from 19.06 ± 0.43 to 34.53 ± 0.24 mg gallic acid equivalents (GAE)/g extract, with the aqueous extract displaying the highest TP_he_C. The current findings underscore the remarkable therapeutic potential of ABAP, which stands out as an abundant source of polyphenols. ABAP boasts not only a diverse array of biological activities but also demonstrates robust antioxidant properties. Experimental data indicate that these polyphenols may hold the key to mitigating oxidative stress, moderating aging processes, and safeguarding against diseases that are intricately linked to oxidative stress, thereby highlighting their multifaceted health benefits.

#### 2.3.6. Total Phenolic Acid Content (TPAC)

Phenolic acids, organic acids characterized by phenolic hydroxyl groups, are ubiquitous in plants and renowned for their exceptional antioxidant properties. Their unique chemical structure, particularly the phenolic hydroxyl group, allows them to effectively scavenge oxygen-free radicals, thereby imparting potent antioxidant and anti-bacterial capabilities. These qualities are vital in food preservation [34,35]. In this study, the TPAC of various extracts of ABAP was evaluated. As presented in Table 1, the TPAC values varied substantially, ranging from 8.97 ± 0.53 to 16.45 ± 0.20 mg caffeic acid equivalents (CAEs)/g extract. Notably, the 80% ethanol extract demonstrated the highest TPAC, closely followed by the methanol extract, aqueous extract, and ethanol extract.

#### 2.3.7. Total Flavonoid Content (TFC)

Flavonoids, a class of natural compounds, exhibit a myriad of biological activities, particularly a robust antioxidant potential, effectively counteracting oxidation mediated by free radicals [36]. Extensive research has demonstrated the exceptional iron ion reduction capabilities and potent scavenging abilities of flavonoids against DPPH and hydroxyl radicals [37,38]. In this study, the TFC of four solvent extracts of ABAP was quantitatively assessed, with values spanning from 4.04 ± 0.21 to 15.44 ± 0.31 mg quercetin equivalents (QE)/g extract, as detailed in Table 1. Notably, the 80% ethanol extract emerged as the richest source of flavonoids, with the highest TFC. This observation offers a plausible rationale for the incongruent results obtained from the previous four qualitative experimental analyses, suggesting that the varying experimental sensitivity might be attributed to the relatively low flavonoid content present in some of the extracts.

#### 2.3.8. Total Tannin Content (TT_an_C), Gallotannin Content (GC), and Condensed Tannin Content (CTC)

Tannins, a ubiquitous and diverse class of polyphenolic compounds found abundantly in nature, particularly in plants, are renowned for their antioxidant and anti-bacterial properties [39,40]. These compounds can be broadly classified into two structural categories: hydrolyzable tannins and condensed tannins, with gallotannin being a prominent member of the hydrolyzable tannin family. To evaluate the tannin content of ABAP extracts obtained using various solvents, we determined key metrics such as TT_an_C, GC, and CTC (Table 1). The TT_an_C values ranged from 16.31 ± 0.20 to 27.25 ± 0.38 mg tannic acid equivalents (TAEs)/g extract, with the aqueous extract displaying the highest TT_an_C. Notably, GC was exclusively detected in the 80% ethanol and aqueous extracts, while CTC remained undetected in all four extracts. These findings offer valuable insights into the discrepancies observed in qualitative analyses, which can be attributed to the varying tannin content in ABAP.

#### 2.3.9. Total Triterpenoid Content (TT_ri_C)

Triterpenoids, a noteworthy class of naturally occurring compounds, boast remarkable structural diversity and a broad spectrum of biological activities, including anti-tumor, anti-inflammatory, and lipid-lowering effects [3]. In this study, the TT_ri_C was meticulously quantified in various extracts of ABAP (Table 1), revealing values that ranged from 12.30 ± 1.05 to 86.85 ± 2.43 mg ginsenoside Re equivalents (GREs)/g extract. Notably, the methanol extract stood out with the highest TT_ri_C, closely trailed by the ethanol extract. These findings echo previous research, which highlights the richness of *Anemone* plants in triterpenoids [2], emphasizing the potential for further exhaustive exploration in this promising area.

In conclusion, this study provides invaluable insights into the potential of ABAP as a treasure trove of bioactive compounds, encompassing carbohydrates, glycosides, monoterpenoids, proteins, phenolics, tannins, and triterpenoids, each endowed with diverse physiological and pharmacological attributes. To fully exploit the benefits of these compounds, further research is imperative, aiming to identify the specific constituents within the monoterpenoid and triterpenoid classes and unravel their underlying mechanisms of action. Such endeavors hold the promise of significantly advancing our understanding of the therapeutic potential of *A. baicalensis* and catalyzing the development of innovative treatments for a plethora of diseases.

### 2.4. Antioxidant Activity In Vitro

#### 2.4.1. 1,1-Diphenyl-2-Picrylhydrazyl Radical (DPPH) and 2,2′-Azino-Bis(3-Ethylbenzothiazoline-6-Sulfonicacid) Diammonium Salt (ABTS)

Currently, the most prevalent and efficient approach to assessing antioxidant activity revolves around evaluating its efficacy in scavenging free radicals. Two extensively utilized in vitro methods for this purpose are the DPPH and ABTS radical scavenging assays. DPPH, being fat-soluble, and ABTS, being water-soluble, make them ideally suited for screening and evaluating natural antioxidants [41,42]. Our experimental study thoroughly examined the free radical scavenging capability of various ABAP extracts. In the DPPH assay, both the methanol and 80% ethanol extracts exhibited robust scavenging abilities. Similarly, in the ABTS assay, the extracts of aqueous, 80% ethanol, and methanol all demonstrated significant ABTS scavenging activity (Table 2). Notably, among the *Anemone* species tested, *Anemone cathayensis* and *Anemone taipaiensis* distinguished themselves for their potent DPPH scavenging capabilities [7,13], which aligns perfectly with our experimental observations.

#### 2.4.2. Hydroxyl Radicals and Superoxide Radicals

Evaluating the antioxidant potential of samples through hydroxyl radical and superoxide radical experiments is a pivotal method. Our study has unveiled that the methanol extract of ABAP exhibited the highest level of scavenging activity against both hydroxyl radicals and superoxide radicals, as evident in Table 2. This finding resonates with previous research, which highlighted the capability of *Anemone raddeana* to scavenge these radicals [43]. Consequently, our results reinforce the antioxidant properties of ABAP methanol extract, adding further credence to its potential health benefits.

#### 2.4.3. Ferric Reducing Antioxidant Power (FRAP) and Cupric Reducing Antioxidant Capacity (CUPRAC)

Evaluating the antioxidant capacity of a sample, a crucial step in the discovery of therapeutic agents, frequently involves assessing its proficiency in reducing iron and copper ions. Two prevalent methods for this assessment are the FRAP and CUPRAC assays. The FRAP assay operates under acidic conditions, whereas the CUPRAC assay functions under neutral conditions, mirroring the physiological environment more closely. Both methods are renowned for their simplicity and cost-effectiveness [44,45]. Notably, the results from FRAP assays positively correlate with those from DPPH assays, and our study has revealed that the methanol extract possesses the most robust ability to reduce iron ions (Table 3). Furthermore, the CUPRAC results demonstrate that both the 80% ethanol extract and the methanol extract possess notable abilities to reduce copper ions (Table 3). Our findings align seamlessly with the previous literature, emphasizing the formidable reducing power of *Anemone taipaiensis* [13].

#### 2.4.4. Metal Chelation

Assessing the metal ion chelating capability of a sample is imperative for understanding its antioxidant potential. The presence of iron and copper ions can accelerate the Fenton reaction, exacerbating oxidative stress within biological systems [46]. Research emphasizes that an abundance of Fe^2+^ in the body can precipitate oxidative damage and is frequently implicated in neurological disorders, such as Alzheimer’s and Parkinson’s disease. The chelation of Fe^2+^ can mitigate the production of ROS and subsequent oxidative damage [47]. Our study has demonstrated that extracts of methanol and 80% ethanol possess robust Fe^2+^ chelating abilities. Additionally, extracts of 80% ethanol, aqueous, and methanol exhibit noteworthy Cu^2+^ chelating capabilities (Table 3). These discoveries highlight the promising potential of *A. baicalensis* as a natural source of chelating agents.

#### 2.4.5. Hydrogen Peroxide (H_2_O_2_) and Singlet Oxygen

H_2_O_2_ is a potent oxidizing agent and a by-product of human metabolism. In physiological equilibrium, the levels of H_2_O_2_ are meticulously maintained through a delicate balance between its generation and elimination within cells. However, disruptions in this delicate balance can elicit cell apoptosis and potentially contribute to the onset of cancer and other severe health complications. Thus, the normal metabolic functioning of human cells is intricately intertwined with the preservation of balanced H_2_O_2_ levels [48]. Our research reveals that extracts of aqueous and methanol possess notable H_2_O_2_ scavenging abilities (Table 4). Conversely, singlet oxygen, another ROS that can inflict cellular damage, was found to be less effectively scavenged by the tested extracts (Table 4). It is noteworthy that there exists a scarcity of literature documenting the singlet oxygen scavenging abilities of plants within the same genus, thereby emphasizing the urgency for additional research in this domain. Singlet oxygen, being a pivotal ROS, holds a crucial position in the physiology of living organisms. Consequently, the investigation of its properties carries scientific weight, and the experimental outcomes obtained serve as a cornerstone for embarking on singlet oxygen experiments specifically tailored to this genus of plants.

#### 2.4.6. β-Carotene Bleaching

Upon ingestion, the human body has the ability to convert *β*-carotene into vitamin A, which plays a crucial role in protecting against free radical damage. Furthermore, *β*-carotene functions as an antioxidant, mitigating the detrimental effects of oxidative stress by minimizing lipid oxidation [49]. However, *β*-carotene, a widely used polyene colorant, is prone to oxidation, resulting in the loss of its characteristic yellow hue. Antioxidants can effectively hinder the oxidation of *β*-carotene, thereby delaying its discoloration. The antioxidant potential of a substance can be evaluated by monitoring the reduction in *β*-carotene’s absorbance over time. In our study, we employed the *β*-carotene bleaching assay to assess the antioxidant capabilities of various ABAP extracts. Our results indicated that the methanol extract exhibited robust antioxidant properties, albeit slightly less potent than synthetic antioxidants such as butylated hydroxytoluene and tertiary butylhydroquinone (Figure S1). Conversely, the ethanol and 80% ethanol extracts demonstrated moderate antioxidant capacity (Table 4). These findings suggest that *A. baicalensis* may serve as a promising natural source of antioxidants, capable of inhibiting oxidation processes and enhancing food preservation during storage.

#### 2.4.7. Hypochlorous Acid (HClO) and Nitric Oxide (NO)

HClO is a potent oxidant that holds a crucial role in safeguarding the body against pathogen invasion. Nevertheless, an overabundance of HClO can disrupt the organism’s delicate oxidative balance, leading to oxidative damage [50]. Our experimental investigation revealed that none of the four extracts derived from ABAP demonstrated the capability to scavenge HClO (Table 4). Furthermore, there is a scarcity of literature reporting on the ability of plants belonging to the same genus to neutralize HClO. Conversely, NO is a vital signaling molecule in biological cells, overseeing numerous cellular functions, engaging in the physiological processes of the cardiovascular and immune systems, and modulating biological signals. NO plays a central role in maintaining bodily homeostasis and regulating specific physiological functions [51]. Our findings demonstrate that the methanol and 80% ethanol extracts exhibited significant NO scavenging abilities (Figure S2). Previous studies have documented the inhibitory effect of the aerial part of *Anemone hupehensis* on NO production [16]. These results imply that *A. baicalensis* may harbor natural compounds with the potential to assist in regulating NO levels within the body.

Our research findings highlight the significant potential of ABAP as a rich source of natural compounds with potent antioxidant activity, particularly evident in its methanol extract. Notably, this extract displayed robust antioxidant capacity, as attested by its high TT_ri_C, TCC, TMC, TP_he_C, TPAC, TFC, and TT_an_C values. It also exhibited remarkable scavenging abilities against diverse ROS, including DPPH, ABTS, hydroxyl radicals, superoxide radicals, H_2_O_2_, and NO, while yielding the highest FRAP and CUPRAC values. Furthermore, it possessed strong metal chelating ability and inhibited *β*-carotene bleaching. Encouraged by these promising results, we aim to further intensify our research efforts, with a primary focus on the methanol extract. Our immediate plans include investigating the chemical composition of this extract to pinpoint the specific compounds responsible for its potent antioxidant properties. Additionally, we aim to conduct oil stability tests and in vivo experiments to substantiate its antioxidant efficacy and evaluate its safety for potential therapeutic applications.

### 2.5. UHPLC-MS Analysis

In this research, we analyzed the chemical makeup of the methanol extract of ABAP using ultrahigh-performance liquid chromatography-electrospray ionization-quadrupole-time of flight-mass spectrometry (UHPLC-ESI-Q-TOF-MS) technology. By meticulously matching molecular and fragment ions with reliable reference data, the sources of the reference data are derived from the published literature (please refer to the analysis process of each peak for further details) and reputable public databases, including the Human Metabolome Database (https://hmdb.ca/, accessed on 25 July 2024) and MassBank (https://www.massbank.jp/, accessed on 25 July 2024). We successfully identified a total of 39 bioactive substances (Table 5). The structures of these identified compounds are visually presented in Figure S3. Our UHPLC-MS findings obtained in positive-ion mode are clearly illustrated in Figure S4, and for a more comprehensive understanding, the MS and MS/MS spectra results are included in the Supplementary Material (Figures S5–S92).

The chemical characterization of the methanol extract of ABAP provides profound insights into its diverse compositional makeup. Notably, many of the identified compounds have been previously isolated from plants of the same genus, and they have been documented to play pivotal roles in antioxidant activities. These findings significantly contribute to understanding the overall efficacy of ABAP as a medicinal plant. This comprehensive analysis lays solid groundwork for advancing future research in this field, enabling the targeted investigation of specific compounds responsible for the observed biological effects. Peak 1 presented an *m*/*z* of 262.1223, with a fragment ion at *m*/*z* of 216.1185 after the elimination of a −CO group, leading to the identification of biotin [52]. The mass spectrum of peak 2 displayed an ion at *m*/*z* 280.1326, and its MS^2^ spectrum showed a fragment at *m*/*z* 262.1230. Peak 3 appeared at *m*/*z* 276.1373, with MS^2^ ions at *m*/*z* 294.1489 [M+NH_4_]^+^ and 132.0983 [M−C_6_H_13_N_2_O_2_+H]^+^, identified as *l*-saccharopine [53]. Peak 4, observed at *m*/*z* 328.1316, was characterized as *N*-fructosyl phenylalanine, with MS^2^ ions at *m*/*z* 310.1224, 282.1275, and 250.1597, indicating the loss of –OH, –COOH, and –C_6_H_5_, respectively [54]. Peak 5 at *m*/*z* 188.0658 exhibited a fragment ion at *m*/*z* 100.0729, indicating losses of −C_5_H_5_, and was thus determined to be *l*-phenylalanine [55]. Peak 6, a [M+NH_4_]^+^ ion at *m*/*z* 144.0767, was suggested to be thymine, with MS^2^ ions at *m*/*z* 127.0348 [M+H]^+^ and 112.9525 [M−CH_3_+H]^+^ corresponding to the literature [56]. Peak 7 was identified as chlorogenic acid at *m*/*z* 355.0952, with characteristic fragment ions at *m*/*z* 377.0768 [M+Na]^+^, 163.0350 [C_9_H_7_O_3_]^+^, and 135.0406 [C_8_H_7_O_2_]^+^ [57]. Peak 8 at *m*/*z* 355.0953, with MS^2^ ions at *m*/*z* 192.1344 [M−C_9_H_7_O_3_+H]^+^, 163.0350 [C_9_H_7_O_3_]^+^, and 135.0406 [C_8_H_7_O_2_]^+^, was tentatively identified as 4-*O*-caffeoylquinic acid based on the literature [58]. Peak 9 displayed [M+NH_4_]^+^ at *m*/*z* 374.1368 and produced significant fragment ions at *m*/*z* 194.1137 [M−C_6_H_11_O_5_+H]^+^ and 163.0347 [C_6_H_11_O_5_]^+^, characteristic of 1-*O*-feruloyl-*β*-*d*-glucose [59]. Peak 10 had [M+NH_4_]^+^ at *m*/*z* 420.1781 and yielded significant fragment ions at *m*/*z* 295.0930 [M−C_7_H_7_O]^+^ and 149.0566 [C_5_H_9_O_5_]^+^, characteristic of benzyl *β*-primeveroside [60].

Peak 11 had an *m*/*z* of 355.0959, with MS^2^ ions at *m*/*z* 192.1345 [M−C_9_H_7_O_3_+H]^+^ and 163.0348 [C_9_H_7_O_3_]^+^, tentatively identified as 1-*O*-caffeoylquinic acid based on the literature [61]. Peak 12, with an *m*/*z* of 339.1008, had MS^2^ ions at *m*/*z* 192.1346 [M−C_9_H_7_O_2_+H]^+^ and 163.0346 [C_9_H_7_O_3_]^+^, and was tentatively characterized as either 4-*p*-coumaroylquinic acid or 3-*p*-coumaroylquinic acid [62]. Peak 13 at *m*/*z* 369.1107 was proposed as 3-*O*-feruloylquinic acid, with the main MS^2^ ions at *m*/*z* 194.1132 and 177.0506 corresponding to the loss of –C_7_H_11_O_5_+H and –C_7_H_11_O_6_, respectively [63]. Peak 14 had an *m*/*z* of 757.2062, with major fragments, including *m*/*z* 779.1869 [M+Na]^+^, 611.1505 [M−C_6_H_10_O_4_+H]^+^, and 432.2707 [C_12_H_21_O_11_+H]^+^, identified as kaempferol 3-*O*-sophoroside 7-*O*-rhamnoside [64]. Peak 15 exhibited a precursor ion [M+H]^+^ at *m*/*z* 611.1481, generating major fragment ions at *m*/*z* 633.1341 [M+Na]^+^, 465.0949 [C_6_H_10_O_4_+H]^+^, and 303.0447 [M−C_12_H_20_O_9_+H]^+^, corresponding to rutin [65]. Peak 16 had an *m*/*z* of 611.1504, with MS^2^ ions at *m*/*z* 633.1323 [M+Na]^+^, 465.0953 [M−C_6_H_11_O_5_+NH_4_]^+^, and 303.0447 [M−C_12_H_21_O_10_+NH_4_]^+^, identified as kaempferol 3-*O*-sophoroside [66]. Peak 17 showed an *m*/*z* of 463.0786 and was recognized as kaempferol 3-glucuronide based on the characteristic fragment ions at *m*/*z* 287.0492 [M−C_6_H_8_O_6_+H]^+^ and 194.1130 [C_6_H_9_O_7_+H]^+^ [67]. The mass spectrum of peak 18 displayed an ion at *m*/*z* 183.0736, with its MS^2^ spectrum showing fragments at *m*/*z* 155.0432 and 127.0124. Peak 19, with an *m*/*z* of 447.0824, was identified as apigenin 7-glucuronide based on the characteristic fragment ions at *m*/*z* 271.0544 [M−C_6_H_8_O_6_+H]^+^ and 194.1133 [C_6_H_9_O_7_+H]^+^ [68]. Peak 20 had [M+H]^+^ at *m*/*z* 477.0926 and produced significant fragment ions at *m*/*z* 301.0651 [M−C_6_H_8_O_6_+H]^+^ and 194.1131 [C_6_H_9_O_7_+H]^+^, characteristic of diosmetin 7-glucuronide [69].

The mass spectrum of peak 21 revealed an ion at *m*/*z* 679.4984, with its MS^2^ spectrum showing fragments at *m*/*z* 359.2275 and 340.2541. Peak 22 had an *m*/*z* of 943.5076 and MS^2^ ions at *m*/*z* 960.5358 [M+NH_4_]^+^ and 781.4589 [M−C_6_H_10_O_5_+H]^+^, identified as 1-*O*-{3-[(3-*O*-hexopyranosylhexopyranosyl)oxy]-28-oxoolean-12-en-28-yl}hexopyranose [70]. The mass spectrum of peak 23 exhibited an ion at *m*/*z* 905.6643, with its MS^2^ spectrum displaying a fragment at *m*/*z* 453.3372. Peak 24 showed a [M+H]^+^ ion at *m*/*z* 1105.5566, producing a major fragment ion at *m*/*z* 309.2008 [C_12_H_21_O_9_]^+^, characteristic of hederagenin 28-*O*-*β*-*d*-glucopyranosyl-(1-3)-*α*-*l*-rhamnopyranosyl-(1-4)-*β*-*d*-glucopyranosyl-(1-6)-*β*-*d*-glucopyranosyl ester [71]. Peak 25 was identified as pulsatiloside A with an *m*/*z* of 929.4904. The MS^2^ spectrum displayed a connection of *m*/*z* 768.4514 with the loss of M−C_6_H_10_O_6_+NH_4_ [72]. Peak 26, with an *m*/*z* of 1089.5630, was identified as oleanolic acid 28-*O*-*β*-*d*-glucopyranosyl-(1-3)-*α*-*l*-rhamnopyranosyl-(1-4)-*β*-*d*-glucopyranosyl-(1-6)-*β*-*d*-glucopyranosyl ester, featuring fragment ions at *m*/*z* 619.4108 and 455.3442, corresponding to the loss of −C_18_H_30_O_14_+H and −C_24_H_41_O_19_, respectively [73]. The mass spectrum of peak 27 exhibited an ion at *m*/*z* 1237.5969, with its MS^2^ spectrum showing fragments at *m*/*z* 1075.5502 [M−C_6_H_10_O_5_+H]^+^, 929.4957 [M−C_12_H_20_O_9_+H]^+^, and 767.4457 [M−C_18_H_30_O_14_+H]^+^, recognized as leonloside D [74]. Peak 28 was observed at *m*/*z* 1383.6523 and characterized as raddeanoside R18. Its MS^2^ ions at m/z 1059.5586 and 913.5021 indicated the loss of –C_12_H_20_O_10_+H and –C_18_H_30_O_14_+H, respectively [75]. Peak 29 had a [M+NH_4_]^+^ ion at *m*/*z* 944.5383, producing major fragment ions at *m*/*z* 340.2525 [C_13_H_23_O_10_+H]^+^ and 309.1122 [C_12_H_21_O_9_]^+^, characteristic of cussonoside B [76]. Peak 30 was detected at *m*/*z* 1400.6834 with fragment ions at *m*/*z* 340.2531 [C_13_H_23_O_10_+H]^+^ and 181.1181 [M−C_59_H_94_O_25_+H]^+^, assigned as hederacolchiside F [77].

Peak 31 was suggested as anhuienoside E by matching fragment ions (*m*/*z* 944.5346 [M−C_12_H_20_O_9_+NH_4_]^+^ and *m*/*z* 340.2530 [C_13_H_23_O_10_+H]^+^) with a reference [78]. Peak 32 at *m*/*z* 1384.6890 had MS^2^ ions at *m*/*z* 1076.5832 [M−C_12_H_20_O_9_+NH_4_]^+^, 516.2719 [C_19_H_31_O_16_+H]^+^, and 309.1126 [C_12_H_21_O_9_]^+^, tentatively assigned as hederacolchiside E [79]. Peak 33 registered an *m*/*z* of 1238.6314 and exhibited fragment ions at *m*/*z* 340.2538 [C_13_H_23_O_10_+H]^+^ and 295.2222 [C_11_H_19_O_9_]^+^, presumptively identified as hederacoside C [77]. Peak 34 displayed a [M+NH_4_]^+^ ion at *m*/*z* 1238.6362, with its main fragment ions at *m*/*z* 369.1878 [C_13_H_21_O_12_]^+^ and 348.2680 [C_12_H_21_O_10_+Na]^+^. These findings were characteristic of raddeanoside R14 [80]. Peak 35 (*m*/*z* 959.5013) was recognized as 3-*O*-*β*-*d*-glucopyranosyl-(1-2)-*β*-*d*-glucopyranosyl-(1-6)-*β*-*d*-galactopyranosyl-hederagenin, based on the typical fragment ions at *m*/*z* 981.4881 [M+Na]^+^, 797.4541 [M−C_6_H_11_O_6_+NH_4_]^+^, and 455.3442 [M−C_18_H_31_O_16_]^+^ [81]. The mass spectrum of peak 36 exhibited an ion at *m*/*z* 274.2677, with its MS^2^ spectrum showing a fragment at *m*/*z* 127.0123. Peak 37 showed an ion at *m*/*z* 318.2931, with its MS^2^ spectrum displaying a fragment at *m*/*z* 274.2691. Peak 38 had an *m*/*z* of 930.5239 and gave fragment ions at *m*/*z* 478.3288 and 460.3174, which correlated with the loss of −C_17_H_29_O_14_ and −C_30_H_46_O_3_+NH_4_, suggesting that peak 38 was raddeanoside R13 [82]. The precursor ion [M+H]^+^ of peak 39 appeared at *m*/*z* 677.3609, with its main fragment ions at *m*/*z* 694.3916 [M+NH_4_]^+^, 497.3011 [M−C_6_H_11_O_6_]^+^, and 353.2626 [C_21_H_35_O_3_+NH_4_]^+^, corresponding to gingerglycolipid A [83]. Peak 40 at *m*/*z* 573.2913 had MS^2^ ions at *m*/*z* 555.2849 [M−OH]^+^ and 537.2768 [M−OH−H_2_O]^+^, identified as 1-palmitoylglycerophosphoinositol [84]. The main fragment ions of peak 41 (*m*/*z* 302.2983) were at *m*/*z* 230.8841 [C_13_H_28_NO_2_]^+^, 127.0124 [C_9_H_19_]^+^, and 100.0728 [C_7_H_15_+H]^+^, assigned as sphinganine [85]. Peak 42 (*m*/*z* 279.2252) was recognized as linolenic acid based on the typical fragment ions at *m*/*z* 236.1960 [C_15_H_24_O_2_]^+^ and 183.0740 [C_11_H_19_O_2_]^+^ [86]. The precursor ion [M+H]^+^ of peak 43 appeared at *m*/*z* 277.2089, with its main fragment ion at *m*/*z* 155.0428 [C_9_H_14_O_2_+H]^+^, corresponding to stearidonic acid. Peak 44 exhibited a [M+Na]^+^ peak at *m*/*z* 277.2090, which produced primary fragment ions at *m*/*z* 196.9607 [C_14_H_27_+H]^+^, 140.1148 [C_10_H_9_+H]^+^, and 130.1559 [C_7_H_13_O_2_+H]^+^. These fragment ions are indicative of palmitoleic acid [87].

### 2.6. Stability of Methanol Extract

**Figure 3:**
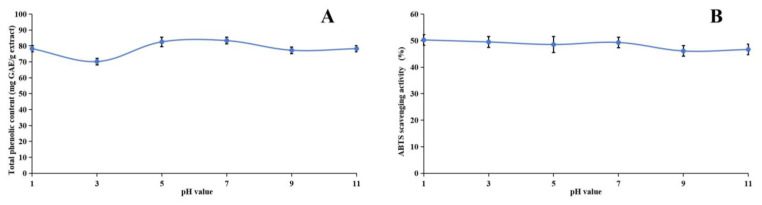
Assessment of pH stability of methanol extract of *Anemone baicalensis* aerial part using TP_he_C (**A**) and ABTS (**B**) assays. TP_he_C: total phenolic content; ABTS: 2,2′-Azino-*bis*(3-ethylbenzothiazoline-6-sulfonicacid) diammonium salt.

**Figure 4:**
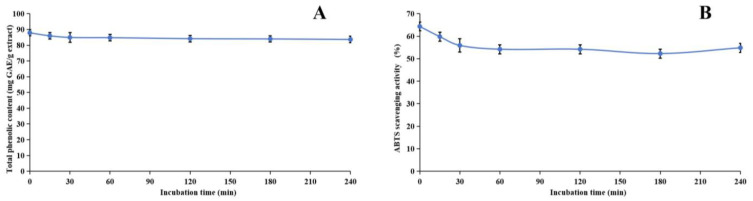
Assessment of thermal stability of methanol extract of *Anemone baicalensis* aerial part using TP_he_C (**A**) and ABTS (**B**) assays. TP_he_C: total phenolic content; ABTS: 2,2′-Azino-*bis*(3-ethylbenzothiazoline-6-sulfonicacid) diammonium salt.

**Figure 5:**
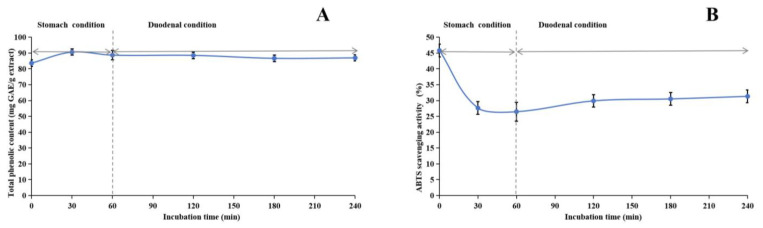
Assessment of in vitro simulated human digestive system on stability of methanol extract of *Anemone baicalensis* aerial part using TP_he_C (**A**) and ABTS (**B**) assays. TP_he_C: total phenolic content; ABTS: 2,2′-Azino-*bis*(3-ethylbenzothiazoline-6-sulfonicacid) diammonium salt.

Our research investigated the stability of the methanol extract of ABAP and its antioxidant capabilities through a comprehensive series of experiments. Figure 3, Figure 4 and Figure 5 illustrate the results of these experiments. We observed that the TP_he_C and ABTS radical scavenging activity of the extract remained relatively constant across various pH levels. Specifically, the TP_he_C value peaked at pH 7 and displayed a slight decrease when the pH was either increased or decreased. Conversely, the ABTS scavenging activity showed a gradual reduction as the pH increased. This decrease can be attributed to the stronger alkalinity negatively impacting the acidic environment necessary for the ABTS assay.

During the heating process, we noticed a significant decline in both the TP_he_C value and the ABTS scavenging activity of the extract within the initial 30 min. Following this initial period, both parameters remained relatively stable. This observation is readily explainable, primarily due to the presence of phenolic acids, such as chlorogenic acid, in the methanol extract. Chlorogenic acid is inherently heat-sensitive and prone to degradation [88], whereas other phenolic compounds present in the extract exhibit greater thermal stability. Consequently, despite the degradation of chlorogenic acid, the overall TP_he_C value and ABTS scavenging activity remain largely unchanged, indicating the stability of the remaining phenolic components.

Regarding stability experiments simulating the human digestive system in vitro, the TP_he_C value of the methanol extract initially increased and subsequently declined over time. This trend suggests that the gastric environment initially facilitates the dissolution of phenolic compounds. However, the subsequent introduction of gastric acid, pepsin, trypsin, pancreatin, and bile likely impacted the extract, resulting in a gradual reduction in the TP_he_C value. Notably, the ABTS radical scavenging activity of the extract underwent a significant decrease under gastric conditions but remained relatively consistent under duodenal conditions. While ABTS is generally stable under acidic conditions, its stability may be compromised in excessively acidic environments, potentially leading to decomposition or modification that could undermine its efficacy as a free radical scavenger. Nevertheless, our stability studies revealed that the antioxidant constituents of the methanol extract of ABAP demonstrated remarkable resilience, indicating their potential to retain effectiveness under various physiological conditions.

### 2.7. Oxidative Stability of Oils

Frying, a ubiquitous and highly favored cooking technique among consumers, offers distinct sensory qualities. However, the extreme temperatures involved can induce chemical alterations in the oils used, ultimately affecting the shelf life and overall quality of fried products. To mitigate these issues, synthetic antioxidants are often employed, albeit with potential risks and hazards. Given the vast demand for edible oils, identifying safe and natural antioxidants has become imperative. A common strategy to assess the primary oxidation status of oils involves evaluating peroxide and acidity levels, alongside metrics such as K_232_ and K_270_ values, which monitor both primary and secondary lipid oxidation processes [89].

**Figure 6:**
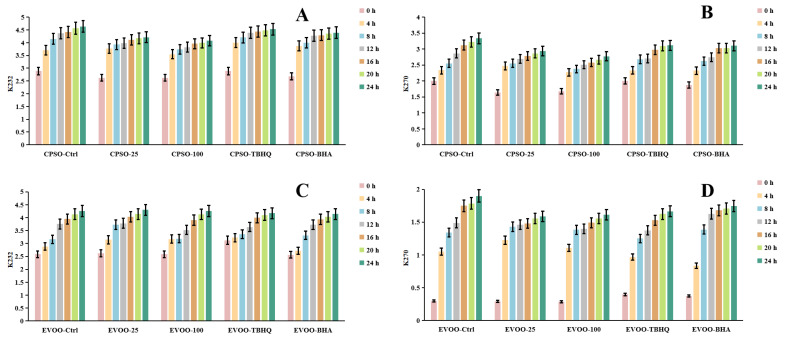
Changes in conjugated dienes (K_232_) and trienes (K_270_) levels in CPSO (**A**,**B**) and EVOO (**C**,**D**) supplemented with synthetic antioxidants, TBHQ and BHA, and varying concentrations of methanol extract of *Anemone baicalensis* aerial part at 160 °C. CPSO: cold-pressed sunflower oil; EVOO: extra virgin olive oil; TBHQ: tertiary butylhydroquinone; BHA: butyl hydroxyanisole.

**Figure 7:**
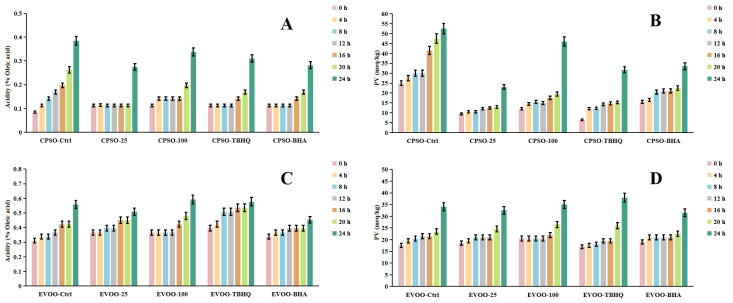
Changes in acidity values and PV levels in CPSO (**A**,**B**) and EVOO (**C**,**D**) supplemented with synthetic antioxidants, TBHQ and BHA, and varying concentrations of methanol extract of *Anemone baicalensis* aerial part at 160 °C. CPSO: cold-pressed sunflower oil; EVOO: extra virgin olive oil; PV: peroxide values; TBHQ: tertiary butylhydroquinone; BHA: butyl hydroxyanisole.

In our research, we employed diverse evaluation techniques to assess the efficacy of various antioxidants. Specifically, we investigated the impact of incorporating varying concentrations of a methanol extract into extra virgin olive oil (EVOO). Our findings revealed a significant antioxidant effect in the K_232_ and K_270_ assays, with the methanol extract outperforming EVOO-25 and demonstrating even greater potency in EVOO-100. However, in the peroxide and acidity assays, the results diverged, with EVOO-100 exhibiting superior antioxidant effects compared to EVOO-25 (Figure 6 and Figure 7). These observations were consistent when using cold-pressed sunflower oil (CPSO) as the test substrate. Further UHPLC-MS analysis identified rutin as a primary constituent of the methanol extract, a compound renowned for its potent antioxidant properties [90]. This finding explains the stability-enhancing effects observed in the oils treated with the methanol extract. In conclusion, the primary objective of conducting the oil stability experiment was to evaluate the antioxidant prowess of the methanol extract. This assessment underscores the critical role that the extract’s antioxidant capabilities play in determining its medicinal value, particularly in its ability to safeguard oils from oxidative degradation.

### 2.8. Oral Acute Toxicity

In our research, we conducted an oral acute toxicity study by administering a single dose of 2000 mg/kg of the methanol extract of ABAP to a group of mice. Our observations revealed that none of the 20 tested mice experienced any adverse effects or succumbed within the 24 h period, indicating that the methanol extract is devoid of highly toxic components that could potentially cause mortality in mice. This finding emphasizes the absence of acute toxicity associated with the extract. Furthermore, a separate study has reported that oral administration of *Anemone raddeana* total saponins to mice at doses of 100 mg/kg and 200 mg/kg over a 28 d period did not result in any toxicity, further reinforcing the safety profile of the compounds under investigation [82]. Collectively, these findings provide compelling evidence that *A. baicalensis* is relatively safe for use in mice, albeit with the caveat that further comprehensive safety evaluations are necessary to fully assess its potential risks and benefits in various applications. Our study serves as foundational data that will inform future toxicological evaluations and dose optimization in subsequent experiments involving rats and potentially non-human primates, where necessary adjustments and validations will be undertaken.

### 2.9. Gastric Protective Activity

The stomach plays a crucial role in normal physiological processes, as it is responsible for secreting stomach acid and digestive enzymes and serves as a vital repository and organ for food digestion in both humans and animals. One of the most common conditions affecting the stomach is gastric ulcer, a disease with multiple causes, including *Helicobacter pylori* infection, unhealthy dietary habits, mental stress, medication use, and other factors. The etiology of gastric ulcers is complex and multifaceted, with oxidative damage playing a significant and pivotal role among the contributing factors. Treatment of gastric ulcers often involves the administration of proton pump inhibitors and histamine H_2_ receptor antagonists, which are proven therapeutic options. However, prolonged use of these medications may increase the risk of developing gastric polyps, tumors, osteoporosis, and other health concerns [91]. Additionally, gastric ulcer protectants frequently carry side effects, emphasizing the pressing need for the development of natural drugs that exhibit fewer adverse reactions and improved safety profiles. The pursuit of such drugs is essential to addressing the burden of gastric ulcers and ensuring better health outcomes for patients.

Both publications highlight that pre-protective studies of anti-ulcer drugs can reveal their potential mechanisms of action and deepen comprehension of the pathological and physiological underpinnings of gastric ulcers, providing a scientific foundation for the development of novel treatment strategies [92,93]. Anhydrous ethanol as an inducer in reports emphasizes its credibility and relevance, which informed our choice of methodology [93]. Therefore, anhydrous ethanol was selected as the model inducer.

To evaluate the therapeutic potential of the methanol extract of ABAP in managing gastric ulcers, we conducted a series of experiments on rats. A total of 40 rats were randomly divided into 5 groups, each comprising 8 rats, and were subjected to distinct oral treatment protocols. The control group (group I) received 0.5% carboxymethylcellulose sodium as a placebo. The positive control group (group II) was administered omeprazole, a standard treatment for gastric ulcers, at a dosage of 30 mg/kg body weight (BW). The model group (group III) was given anhydrous ethanol in combination with 0.5% carboxymethylcellulose sodium to induce gastric ulceration. The experimental groups included a low-dose group (group IV), which was treated with the methanol extract at a dosage of 200 mg/kg BW, and a high-dose group (group V), which received the extract at a dosage of 400 mg/kg BW. By employing this experimental design, we aimed to compare the efficacy of the methanol extract against the standard treatment and assess its ability to protect against gastric ulceration induced by anhydrous ethanol. We will incorporate serum biochemical indicators and biomarker detection to accurately assess the extract’s effects on animal health.

**Figure 8:**
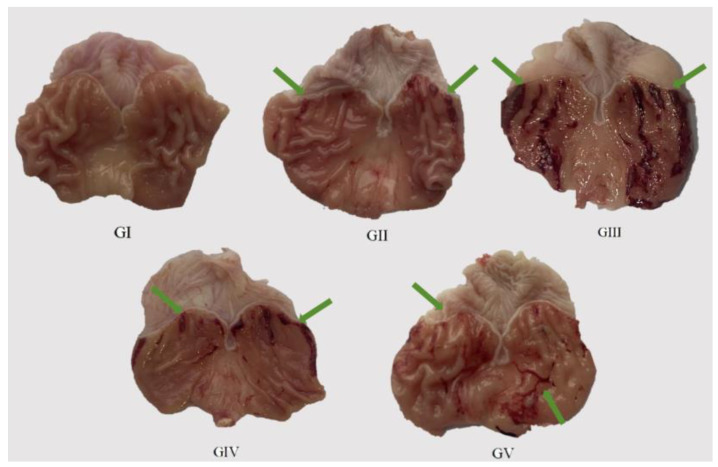
Macroscopic images of representative gastric tissues from each treatment group. Green arrow: bleeding bands and punctate ulcers.

We administered the methanol extract to the rats daily for seven consecutive days via gavage, followed by an oral dose of anhydrous ethanol. The subsequent assessment of the excised gastric mucosa damage in each group offered a direct glimpse into the health status of the gastric tissue. The results of the histological examination of the gastric tissue morphology for each group of rats are presented in Figure 8, while the ulcer index and ulcer inhibition rate for each group are detailed in Table 6. Upon histological observation, it was evident that the surface layer of the gastric mucosa in the model group rats exhibited substantial damage, characterized by multiple dark red bleeding bands and punctate ulcers. In stark contrast, the rats in the positive control group demonstrated no significant damage to their gastric mucosa, mirroring the observations in the control group. Notably, both the rats in the high-dose group (400 mg/kg) and the low-dose group (200 mg/kg) showed a relatively reduced degree of gastric mucosal damage, with only a small number of ulcer bleeding points visible. Remarkably, the ulcer inhibition rates in both the low-dose and high-dose groups increased proportionally with the dosage, achieving 65.74% and 76.12%, respectively. This finding highlights the pre-protective effect of the methanol extract on rat gastric ulcers, with its efficacy positively correlated with the dosage administered. Consequently, the methanol extract effectively mitigated the damaging effects of anhydrous ethanol on the rat gastric mucosa.

**Figure 9:**
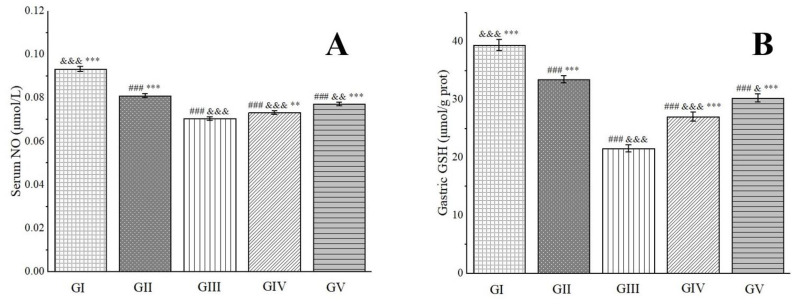
Effects of *Anemone baicalensis* aerial part on serum NO (**A**) and gastric GSH (**B**) in rats with gastric ulcer. NO: nitric oxide; GSH: glutathione. Significantly different from the group I at ### *p* < 0.001. Significantly different from the group II at ^&^ *p* < 0.05, ^&&^ *p* < 0.01, and ^&&&^ *p* < 0.001. Significantly different from the group III at ** *p* < 0.01 and *** *p* < 0.001.

NO possesses robust antioxidant properties and exerts potent protective effects on the gastric mucosa. It directly dilates blood vessels by relaxing vascular smooth muscle, thereby enhancing gastric mucosal blood flow and improving the vascular permeability of damaged tissue [94]. Glutathione (GSH), another vital antioxidant, effectively scavenges H_2_O_2_ and oxygen-free radicals, prevents lipid peroxidation in tissues, and collaborates with antioxidant enzymes to provide a protective barrier [95]. Experimental findings have revealed that, in comparison to group III, the NO levels in groups IV and V were significantly elevated. Furthermore, the GSH levels in rats from groups IV and V increased by 20.23% and 28.73%, respectively, nearing levels comparable to those in group II (Figure 9). These results suggest that pre-treatment with the methanol extract exhibited gastric protective effects comparable to those of omeprazole. This outcome is primarily attributed to the presence of chlorogenic acid in the methanol extract, which possesses the capability to restore GSH levels in ethanol-induced gastric ulcers in mice [96]. Additionally, rutin, another component found in the methanol extract, exerts a protective effect against gastric mucosal injury induced by gastric ischemia–reperfusion in rats. This gastric protective effect of rutin is intricately linked to the nitric oxide synthase/NO pathway and its potent antioxidant activity [97].

**Figure 10:**
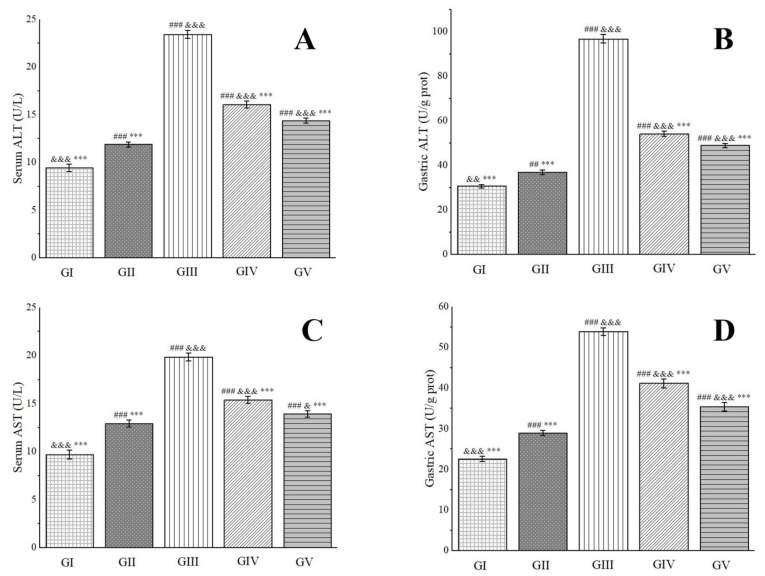
Effects of *Anemone baicalensis* aerial part on serum ALT (**A**), gastric ALT (**B**), serum AST (**C**), and gastric AST (**D**) in rats with gastric ulcer. ALT: alanine aminotransferase; AST: aspartate aminotransferase. Significantly different from the group I at ^##^ *p* < 0.01 and ^###^ *p* < 0.001. Significantly different from the group II at ^&^ *p* < 0.05, ^&&^ *p* < 0.01, and ^&&&^ *p* < 0.001. Significantly different from the group III at *** *p* < 0.001.

Alanine aminotransferase (ALT), predominantly located in the liver, heart, and skeletal muscle, undergoes an elevation in blood levels subsequent to damage or necrosis of liver cells or certain tissues [98]. Aspartate aminotransferase (AST), a mitochondrial enzyme, can be influenced by ethanol metabolism, which elicits oxidative stress and lipid peroxidation reactions. These reactions, consequently, impair mitochondria, leading to a surge in AST levels [99]. As cells sustain damage or necrosis, both ALT and AST levels increase, rendering them valuable biomarkers for assessing the extent of gastric tissue damage. As depicted in Figure 10, rats in group IV and group V exhibited significantly lower serum and gastric tissue levels of ALT and AST compared to those in group III. Notably, in gastric tissue, the ALT activity in group IV and group V rats was 56.00% and 50.55% of that observed in group III, respectively. Similarly, AST activity was 76.34% and 65.63% of group III’s levels, respectively. These findings indicate that a high dose of the methanol extract demonstrates a superior therapeutic effect on ethanol-induced gastric ulcers.

**Figure 11:**
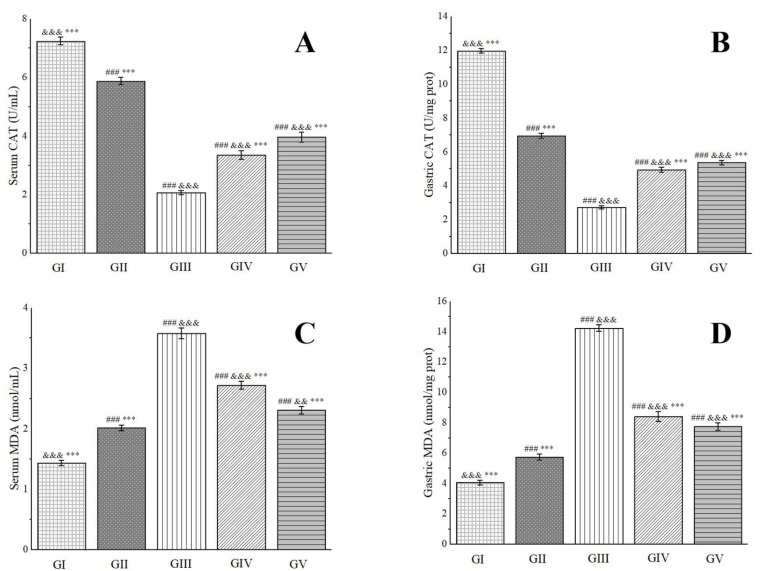
Effects of *Anemone baicalensis* aerial part on serum CAT (**A**), gastric CAT (**B**), serum MDA (**C**), and gastric MDA (**D**) in rats with gastric ulcer. CAT: catalase; MDA: malondialdehyde. Significantly different from the group I at ^###^ *p* < 0.001. Significantly different from the group II at ^&&^ *p* < 0.01 and ^&&&^ *p* < 0.001. Significantly different from the group III at *** *p* < 0.001.

Catalase (CAT), a ubiquitous antioxidant enzyme prevalent in living organisms, performs a crucial role in catalyzing the breakdown of H_2_O_2_ into water and oxygen. This process effectively eliminates H_2_O_2_ from the body, thereby safeguarding cells from its detrimental effects. Functioning as a vigilant scavenger within the antioxidant defense system, CAT prevents oxidative damage to membrane proteins and other essential tissues [100]. Our research endeavors have uncovered that the CAT levels in the gastric tissues of rats in group IV and group V underwent significant elevations, with notable increases of 44.85% and 49.18%, respectively, in comparison to group III (Figure 11). Notably, the presence of chlorogenic acid in the methanol extract has been found to rejuvenate CAT levels, exhibiting a protective shield against ethanol-induced gastric ulcers [96].

Malondialdehyde (MDA), the primary by-product of lipid peroxidation in the body, is renowned for its potent toxic effects that can severely disrupt the structure and functionality of cells. Its concentration serves as a reliable indicator of the extent of lipid peroxidation occurring within the body, mirroring the degree of cellular damage inflicted by free radicals [101]. Furthermore, it provides an indirect assessment of gastric damage [102]. In our experimental setup, we observed a pronounced decrease in MDA levels in the gastric tissues of rats belonging to group IV and group V, with reductions of 40.97% and 45.58%, respectively, when compared to group III (Figure 11). Consistent with previous reports, rutin has demonstrated remarkable efficacy in reducing MDA levels in rats afflicted with ethanol-induced gastric ulcers, even at low doses ranging from 20 to 80 mg/kg, underscoring its potent anti-ulcer properties [103]. These cumulative findings suggest that the methanol extract under investigation exerts a protective effect against ethanol-induced gastric ulcer damage in rats.

**Figure 12:**
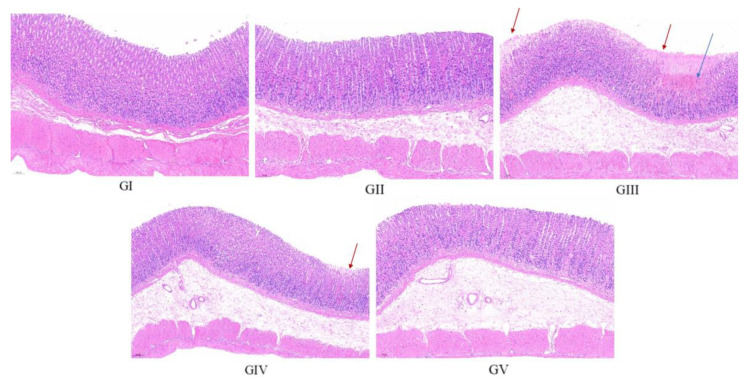
Representative histopathological photos of gastric tissue sections from each group (100× magnification). Red arrow: disorganization of the glandular arrangement; blue arrow: infiltration and congestion of inflammatory cells.

The histopathological analysis findings are presented in Figure 12. In the control group, the gastric mucosa of rats displayed a pristine, undamaged structure, characterized by a neatly arranged pattern of gastric pits and glands, devoid of congestion or shedding of upper epidermal cells. Conversely, the gastric mucosal architecture of rats in the model group was visibly compromised, exhibiting disorganization of the glandular arrangement (marked by the red arrow). Furthermore, the submucosal layer revealed infiltration and congestion of inflammatory cells (marked by the blue arrow). In stark contrast, the gastric mucosa of rats in the positive control group remained unscathed, mirroring the pristine state observed in the control group. Upon comparison with the model group, rats in both the low-dose and high-dose treatment groups exhibited a more structured arrangement of gastric mucosal glands, accompanied by reduced infiltration of inflammatory cells and lesser damage. Notably, the high-dose group demonstrated a pre-protective effect comparable to omeprazole, further underscoring its efficacy. These observations align closely with the morphological evaluation of gastric tissue, reinforcing the effectiveness of the treatment in mitigating gastric damage.

In summary, through comprehensive visual inspection and histopathological analysis of rat gastric tissue, coupled with the evaluation of various oxidative stress-related biomarkers in both rat serum and gastric tissue, it has been conclusively demonstrated that the methanol extract of ABAP possesses a remarkable protective effect against ethanol-induced gastric ulcers in rats. This protective effect exhibits a clear dose-dependency, where higher doses yield more pronounced outcomes, approaching the efficacy of omeprazole, a universally acknowledged treatment option. This discovery emphasizes the significance of further extensive research into the therapeutic potential of the methanol extract, which holds immense promise for future applications.

## 3. Material and Methods

### 3.1. Materials

In May 2021, the plant *A. baicalensis* was collected (voucher specimen number: 2021-05-29-001) from Tonghua, situated in Jilin Province of China, at a precise geographical location marked by latitude N 42°0′50.80″ and longitude E 126°12′40.11″, and an elevation of 683.4 m. The authentication of these specimens was carried out by professor Junlin Yu, and the voucher specimen is now preserved within the Herbarium of Tonghua Normal University.

### 3.2. Methods

#### 3.2.1. Qualitative Phytochemical Analysis

A qualitative phytochemical analysis was carried out on fifteen distinct chemical components, adhering strictly to a previously validated methodology [104]. For a comprehensive understanding of the detailed experimental procedures, kindly refer to the Supplementary Materials provided.

#### 3.2.2. Preparation of Different Extracts of ABAP

To investigate the effect of different solvents on the efficacy of extracting active constituents from ABAP, this experiment utilized four specifically chosen polar solvents, namely, methanol, water, ethanol, and 80% ethanol. The extraction protocol adhered strictly to our previously established methodology [104]. For a comprehensive understanding of the detailed experimental procedures, kindly refer to the Supplementary Materials provided.

#### 3.2.3. Quantitative Phytochemical Analysis

A comprehensive quantitative phytochemical analysis was conducted to measure the concentrations of various compounds, such as TCC, TP_ro_C, TMC, TAC, TP_he_C, TPAC, TFC, TT_an_C, CTC, GC, and TT_ri_C. This analysis was carried out using a methodology that was based on previously established and well-documented procedures as referenced in [21,104]. For a detailed outline of the experimental procedures employed, kindly refer to the Supplementary Materials provided.

#### 3.2.4. Antioxidant Activity Assays

A comprehensive set of antioxidant activity assays were performed utilizing diverse methodologies, including DPPH, ABTS, hydroxyl radical, superoxide radical, FRAP, CUPRAC, metal chelation, H_2_O_2_, singlet oxygen, *β*-carotene bleaching, HClO, and NO assays. These assays strictly adhered to the previously established protocols outlined in references [21,104]. For a detailed account of the experimental procedures, kindly refer to the Supplementary Materials provided.

#### 3.2.5. UHPLC-MS Method

The experimental conditions for UHPLC-MS were rigorously followed as described in our previously established method [104], thereby ensuring the precision and reproducibility of the analytical procedure. For a comprehensive understanding of the detailed experimental procedures, kindly refer to the Supplementary Materials provided.

#### 3.2.6. Stability Studies of Methanol Extract

We performed an evaluation of the pH stability, thermal stability, and stability within a gastrointestinal tract model system for the methanol extract of ABAP, adhering strictly to the protocols outlined in our previously established methods [104]. For a comprehensive understanding of the detailed experimental procedures, kindly refer to the Supplementary Materials provided.

#### 3.2.7. Oxidative Stability Studies of Oils

The oxidative stability of EVOO and CPSO was rigorously analyzed in accordance with our previously reported methodologies [104], enabling a precise assessment of their resilience against oxidative degradation. For a comprehensive understanding of the detailed experimental procedures, kindly refer to the Supplementary Materials provided.

#### 3.2.8. Oral Acute Toxicity Study

An acute oral toxicity study was conducted in accordance with the well-established protocols outlined in our previous methods [104], with the aim of evaluating the potential adverse effects of the methanol extract under investigation when administered orally in a single dose, which is to preliminary evaluate the safety of the plant extract and provide a foundation for dose selection in future experiments. For a comprehensive understanding of the detailed experimental procedures, kindly refer to the Supplementary Materials provided.

#### 3.2.9. Gastric Protective Experiments

The methodology outlined in reference [105] is devised to investigate the therapeutic potential of methanol extract in ameliorating ethanol-induced gastric ulcers in rats, offering valuable insights into its protective effects against gastric damage stemming from alcohol consumption. For a comprehensive understanding of the detailed experimental procedures, kindly refer to the Supplementary Materials provided.

#### 3.2.10. Statistical Analysis

To assess the statistical significance of the gathered data, we conducted a rigorous statistical analysis. The outcomes were presented as mean values accompanied by their corresponding standard deviations. To discern significant differences among the various groups, we employed a one-way analysis of variance, followed by post-hoc least significant difference tests and DUNCAN tests. We considered *p*-values of 0.05, 0.01, and 0.001 as indicative of significance, high significance, and very high significance, respectively.

## 4. Conclusions

The traditional medicinal applications of *A. baicalensis*, renowned for its detoxification and insecticidal properties, have been extensively harnessed over time. However, a notable void persists in the realm of research and pharmacological evaluation pertaining to its compositional intricacies. This study aims to conduct a thorough evaluation of the antioxidant properties of ABAP, addressing the current lack of comprehensive understanding in this area. Our initial qualitative analysis unveiled the presence of 12 distinct phytochemicals within ABAP, while subsequent quantitative analysis emphasized the plant’s abundance in various bioactive components. Notably, the activity evaluation shone a spotlight on ABAP exceptional antioxidant capabilities, with the methanol extract emerging as the most potent among the tested extracts. Consequently, this extract was chosen for further in-depth exploration. Upon closer examination of the methanol extract, we uncovered an impressive total of 39 bioactive compounds. To ascertain its potential as an antioxidant agent, we conducted a series of rigorous stability and antioxidant capacity assessments under varying conditions, including exposure to heat, different pH environments, and in vitro digestion. Impressively, the extract demonstrated remarkable stability and sustained antioxidant prowess, even under adverse conditions. Additionally, the oil stability experiment validated its ability to stabilize both EVOO and CPSO, thereby ensuring their preservation and maintaining their quality over time. To validate its efficacy in a biological setting, we performed in vivo antioxidant experiments. The results were compelling, demonstrating that the high-dose methanol extract displayed a notable pre-protective effect against gastric ulcer in rats. This protection was achieved by reducing MDA, ALT, and AST levels while simultaneously increasing GSH, NO, and CAT levels. This exhaustive study not only contributes to the understanding of the composition of *A. baicalensis* but also highlights its promising role as a natural antioxidant, emphasizing its significance in the field. In conclusion, our research highlights the remarkable antioxidant attributes of the methanol extract of ABAP. The discovery of these potent active compounds serves as a cornerstone for advancing our understanding of this plant’s potential therapeutic applications in combating oxidative stress-related diseases. We anticipate that our findings will ignite renewed interest in exploring the antioxidant properties of *A. baicalensis*, ultimately contributing to the development of innovative natural remedies that can enhance human health and wellbeing.

## Figures and Tables

**Table 1:** Total carbohydrate content (TCC), total protein content (TP_ro_C), total monoterpenoid content (TMC), total alkaloid content (TAC), total phenolic content (TP_he_C), total phenolic acid content (TPAC), total flavonoid content (TFC), total tannin content (TT_an_C), gallotannin content (GC), condensed tannin content (CTC), and total triterpenoid content (TT_ri_C) of *Anemone baicalensis* aerial part extracted with different solvents.

Extracting Solvents	TCC (mg GE/g Extract)	TP_ro_C (mg BSAE/g Extract)	TMC (mg LE/g Extract)	TAC (mg BHE/g Extract)	TP_he_C (mg GAE/g Extract)	TPAC (mg CAE/g Extract)	TFC (mg QE/g Extract)	TT_an_C (mg TAE/g Extract)	GC (mg GAE/g Extract)	CTC (mg GAE/g Extract)	TT_ri_C (mg GRE/g Extract)
Methanol	424.65 ± 3.08 ^a^	N.T.	1152.54 ± 19.05 ^d^	4.36 ± 0.03 ^b^	30.92 ± 0.15 ^c^	15.38 ± 0.86 ^a^	13.73 ± 0.47 ^b^	22.72 ± 0.11 ^c^	None	None	86.85 ± 2.43 ^a^
Water	355.38 ± 2.86 ^c^	493.08 ± 3.15 ^a^	1430.19 ± 14.12 ^b^	4.12 ± 0.03 ^c^	34.53 ± 0.24 ^a^	11.62 ± 0.79 ^b^	4.04 ± 0.21 ^d^	27.25 ± 0.38 ^a^	4.33 ± 0.11 ^b^	None	12.30 ± 1.05 ^d^
Ethanol	433.95 ± 5.17 ^a^	N.T.	1260.91 ± 21.39 ^c^	5.71 ± 0.01 ^a^	19.06 ± 0.43 ^d^	8.97 ± 0.53 ^c^	6.14 ± 0.12 ^c^	16.31 ± 0.20 ^d^	None	None	73.20 ± 1.62 ^b^
80% Ethanol	389.32 ± 3.24 ^b^	N.T.	1622.28 ± 15.72 ^a^	3.00 ± 0.00 ^d^	31.57 ± 0.58 ^b^	16.45 ± 0.20 ^a^	15.44 ± 0.31 ^a^	25.93 ± 0.18 ^b^	6.29 ± 0.15 ^a^	None	42.97 ± 0.75 ^c^

^a–d^ Columns with different superscripts indicate a significant difference (*p* < 0.05). N.T. indicates no test. GE: glucose equivalents; BSAE: bovine serum albumin equivalents; LE: linalool equivalents; BHE: berberine hydrochloride equivalents; GAE: gallic acid equivalents; CAE: caffeic acid equivalents; QE: quercetin equivalents; TAE: tannic acid equivalents; GRE: ginsenoside Re equivalents.

**Table 2:** Assessment of the antioxidant potential in *Anemone baicalensis* aerial part through DPPH, ABTS, hydroxyl, and superoxide radical scavenging assays.

Extracting Solvents	DPPH (IC_50_, μg/mL)	ABTS (IC_50_, μg/mL)	Hydroxyl Radicals (%, 2500 μg/mL)	Superoxide Radicals (IC_50_, μg/mL)
Methanol	37.21 ± 1.66 ^c^	71.21 ± 2.05 ^d^	47.28 ± 0.43 ^c^	920.36 ± 3.18 ^b^
Water	93.53 ± 2.35 ^e^	52.36 ± 0.54 ^b^	41.12 ± 1.21 ^d^	>2143 ^c^
Ethanol	99.76 ± 2.45 ^f^	125.03 ± 2.40 ^e^	40.74 ± 1.62 ^d^	>2143 ^c^
80% Ethanol	45.14 ± 0.88 ^d^	58.10 ± 1.36 ^c^	42.15 ± 1.08 ^d^	>2143 ^c^
Trolox *	2.11 ± 0.10 ^a^	3.43 ± 0.11 ^a^	89.18 ± 0.87 ^a^	N.T.
BHT *	9.46 ± 0.22 ^b^	4.48 ± 0.04 ^a^	70.14 ± 1.13 ^b^	N.T.
Curcumin *	N.T.	N.T.	N.T.	78.68 ± 0.82 ^a^

^a–f^ Columns with different superscripts indicate a significant difference (*p* < 0.05). * Used as a standard antioxidant; N.T. indicates no test.

**Table 3:** Assessment of the antioxidant potential in *Anemone baicalensis* aerial part through FRAP, CUPRAC, and metal chelation assays.

Extracting Solvents	TEAC_FRAP_	TEAC_CUPRAC_	Iron Chelation (IC_50_, μg/mL)	Copper Chelation (IC_50_, μg/mL)
Methanol	0.22 ± 0.00 ^b^	0.14 ± 0.00 ^c^	921.30 ± 4.04 ^b^	473.20 ± 4.85 ^d^
Water	0.20 ± 0.00 ^b^	0.13 ± 0.00 ^c^	2019.96 ± 13.63 ^e^	347.42 ± 3.84 ^c^
Ethanol	0.18 ± 0.00 ^c^	0.06 ± 0.00 ^d^	1481.40 ± 11.92 ^d^	1243.17 ± 5.87 ^e^
80% Ethanol	0.21 ± 0.00 ^b^	0.17 ± 0.00 ^b^	1065.22 ±10.94 ^c^	317.04 ± 3.12 ^b^
Trolox *	0.92 ± 0.01 ^a^	0.89 ± 0.02 ^a^	N.T.	N.T.
EDTANa_2_ *	N.T.	N.T.	2.28 ± 0.11 ^a^	31.51 ± 0.59 ^a^

^a–e^ Columns with different superscripts indicate a significant difference (*p* < 0.05). * Used as a standard antioxidant; N.T. indicates no test.

**Table 4:** Assessment of the antioxidant potential in *Anemone baicalensis* aerial part through H_2_O_2_, singlet oxygen, *β*-carotene bleaching, and HClO assays.

Extracting Solvents	H_2_O_2_ (IC_50_, μg/mL)	Singlet Oxygen (%, 2000 μg/mL)	*β*-Carotene Bleaching AAC	HClO (IC_50_, μg/mL)
Methanol	1143.52 ± 6.17 ^c^	15.30 ± 1.29 ^d^	496.45 ± 2.59 ^c^	None
Water	970.74 ± 8.13 ^b^	5.91 ± 0.39 ^e^	443.68 ± 2.12 ^d^	None
Ethanol	1522.10 ± 10.39 ^e^	25.40 ± 1.46 ^c^	482.21 ± 2.29 ^c^	None
80% Ethanol	1393.38 ± 8.05 ^d^	40.20 ± 1.18 ^b^	483.12 ± 2.37 ^c^	None
Trolox *	N.T.	N.T.	N.T.	11.22 ± 0.10 ^a^
Lipoic acid *	N.T.	N.T.	N.T.	24.75 ± 0.78 ^a^
Gallic acid *	30.74 ± 1.07 ^a^	N.T.	N.T.	N.T.
Ferulic acid *	N.T.	89.37 ± 1.02 ^a^	N.T.	N.T.
BHT *	N.T.	N.T.	873.67 ± 3.56 ^a^	N.T.
TBHQ *	N.T.	N.T.	802.70 ± 4.28 ^b^	N.T.

^a–e^ Columns with different superscripts indicate a significant difference (*p* < 0.05). * Used as a standard antioxidant; N.T. indicates no test.

**Table 5:** Compounds identified in methanol extract of *Anemone baicalensis* aerial part.

Peak No.	RT (min)	Identification	Molecular Formula	Selective Ion	Full Scan MS (*m*/*z*)		MS/MS Fragments (*m*/*z*)
					Theory	Measured	
1	0.93	Biotin	C_10_H_16_N_2_O_3_S	[M+NH_4_]^+^	262.1226	262.1223	216.1185
2	1.28	Unknown				280.1326	262.1230
3	1.35	*l*-Saccharopine	C_11_H_20_N_2_O_6_	[M]^+^	276.1321	276.1373	294.1489, 132.0983
4	2.43	*N*-Fructosyl phenylalanine	C_15_H_21_NO_7_	[M+H]^+^	328.1396	328.1316	310.1224, 282.1275, 250.1597
5	4.51	*l*-Phenylalanine	C_9_H_11_NO_2_	[M+Na]^+^	188.0688	188.0658	100.0729
6	6.79	Thymine	C_5_H_6_N_2_O_2_	[M+NH_4_]^+^	144.0773	144.0767	127.034, 112.9525
7	7.10	Chlorogenic acid	C_16_H_18_O_9_	[M+H]^+^	355.1029	355.0952	377.0768, 163.0350, 135.0406
8	7.62	4-*O*-Caffeoylquinic acid	C_16_H_18_O_9_	[M+H]^+^	355.1029	355.0953	192.1344, 163.0350, 135.0406
9	8.95	1-*O*-Feruloyl-*β*-*d*-glucose	C_16_H_20_O_9_	[M+NH_4_]^+^	374.1451	374.1368	194.1137, 163.0347
10	9.08	Benzyl *β*-primeveroside	C_18_H_26_O_10_	[M+NH_4_]^+^	420.1870	420.1781	295.0930, 149.0566
11	9.39	1-*O*-Caffeoylquinic acid	C_16_H_18_O_9_	[M+H]^+^	355.1029	355.0959	192.1345, 163.0348
12	9.80	4-*p*-Coumaroylquinic acid/3-*p*-Coumaroylquinic acid	C_16_H_18_O_8_	[M+H]^+^	339.1080	339.1008	192.1346, 163.0346
13	11.55	3-*O*-Feruloylquinic acid	C_17_H_20_O_9_	[M+H]^+^	369.1185	369.1107	194.1132, 177.0506
14	13.05	Kaempferol 3-*O*-sophoroside 7-*O*-rhamnoside	C_33_H_40_O_20_	[M+H]^+^	757.2191	757.2062	779.1869, 611.1505, 432.2707
15	14.85	Rutin	C_27_H_30_O_16_	[M+H]^+^	611.1612	611.1481	633.1341, 465.0949, 303.0447
16	15.30	Kaempferol 3-*O*-sophoroside	C_27_H_30_O_16_	[M+H]^+^	611.1612	611.1504	633.1323, 465.0953, 303.0447
17	15.97	Kaempferol 3-glucuronide	C_21_H_18_O_12_	[M+H]^+^	463.0876	463.0786	287.0492, 194.1130
18	17.68	Unknown				183.0736	155.0432, 127.0124
19	18.88	Apigenin 7-glucuronide	C_21_H_18_O_11_	[M+H]^+^	447.0927	447.0824	271.0544, 194.1133
20	19.86	Diosmetin 7-glucuronide	C_22_H_20_O_12_	[M+H]^+^	477.1033	477.0926	301.0651, 194.1131
21	22.61	Unknown				679.4984	359.2275, 340.2541
22	25.84	1-*O*-{3-[(3-*O*-Hexopyranosylhexopyranosyl)oxy]-28-oxoolean-12-en-28-yl}hexopyranose	C_48_H_78_O_18_	[M+H]^+^	943.5266	943.5076	960.5358, 781.4589
23	26.05	Unknown				905.6643	453.3372
24	26.37	Hederagenin 28-*O*-*β*-*d*-glucopyranosyl-(1-3)-*α*-*l*-rhamnopyranosyl-(1-4)-*β*-*d*-glucopyranosyl-(1-6)-*β*-*d*-glucopyranosyl ester	C_54_H_88_O_23_	[M+H]^+^	1105.5794	1105.5566	309.2008
25	26.75	Pulsatiloside A	C_47_H_76_O_18_	[M+H]^+^	929.5110	929.4904	768.4514
26	26.95	Oleanolic acid 28-*O*-*β*-*d*-glucopyranosyl-(1-3)-*α*-*l*-rhamnopyranosyl-(1-4)-*β*-*d*-glucopyranosyl-(1-6)*-β*-*d*-glucopyranosyl ester	C_54_H_88_O_22_	[M+H]^+^	1089.5845	1089.5630	619.4108, 455.3442
27	27.61	Leonloside D	C_59_H_96_O_27_	[M+H]^+^	1237.6217	1237.5969	1075.5502, 929.4957, 767.4457
28	28.41	Raddeanoside R18	C_65_H_106_O_31_	[M+H]^+^	1383.6796	1383.6523	1059.5586, 913.5021
29	29.98	Cussonoside B	C_48_H_78_O_17_	[M+NH_4_]^+^	944.5583	944.5383	340.2525, 309.1122
30	31.67	Hederacolchiside F	C_65_H_106_O_31_	[M+NH_4_]^+^	1400.7062	1400.6834	340.2531, 181.1181
31	32.37	Anhuienoside E	C_60_H_98_O_26_	[M+NH_4_]^+^	1252.6690	1252.6458	944.5346, 340.2530
32	32.52	Hederacolchiside E	C_65_H_106_O_30_	[M+NH_4_]^+^	1384.7113	1384.6890	1076.5832, 516.2719, 309.1126
33	32.94	Hederacoside C	C_59_H_96_O_26_	[M+NH_4_]^+^	1238.6534	1238.6314	340.2538, 295.2222
34	33.17	Raddeanoside R14	C_59_H_96_O_26_	[M+NH_4_]^+^	1238.6534	1238.6362	369.1878, 348.2680
35	36.34	3-*O*-*β*-*d*-glucopyranosyl-(1-2)-*β*-*d*-glucopyranosyl-(1-6)-*β*-*d*-galactopyranosyl-hederagenin	C_48_H_78_O_19_	[M+H]^+^	959.5215	959.5013	981.4881, 797.4541, 455.3442
36	37.75	Unknown				274.2677	127.0123
37	38.18	Unknown				318.2931	274.2691
38	39.79	Raddeanoside R13	C_47_H_76_O_17_	[M+NH_4_]^+^	930.5427	930.5239	478.3288, 460.3174
39	41.53	Gingerglycolipid A	C_33_H_56_O_14_	[M+H]^+^	677.3748	677.3609	694.3916, 497.3011, 353.2626
40	41.89	1-Palmitoylglycerophosphoinositol	C_25_H_49_O_12_P	[M+H]^+^	573.3040	573.2913	555.2849, 537.2768
41	42.42	Sphinganine	C_18_H_39_NO_2_	[M+H]^+^	302.3059	302.2983	230.8841, 127.0124, 100.0728
42	45.95	Linolenic acid	C_18_H_30_O_2_	[M+H]^+^	279.2324	279.2252	236.1960, 183.0740
43	47.21	Stearidonic acid	C_18_H_28_O_2_	[M+H]^+^	277.2167	277.2089	155.0428
44	47.60	Palmitoleic acid	C_16_H_30_O_2_	[M+Na]^+^	277.2144	277.2090	196.9607, 140.1148, 130.1559

RT: retention time.

**Table 6:** Ulcer index and ulcer inhibition rate of rats in each group.

Group	Ulcer Index (mm)	Ulcer Inhibition Rate (%)
GI	N.T.	N.T.
GII	13.33 ± 0.83 ^###^	81.55
GIII	72.25 ± 3.43 ***	N.T.
GIV	24.75 ± 1.26 ^###^ ***	65.74
GV	17.25 ± 1.68 ^###^ ***	76.12

Values are expressed as the mean ± standard deviation of the mean (*n* = 8). N.T. indicates no test. Significantly different from group II at ^###^ *p* < 0.001. Significantly different from group GIII at *** *p* < 0.001.

## Data Availability

Data are contained within the article.

## Supplementary Materials

The following supporting information can be downloaded at: https://www.mdpi.com/article/10.3390/molecules29194602/s1, Table S1: Phytochemical analysis of *Anemone baicalensis* aerial part; Figure S1: Alterations in the absorbance of *β*-carotene in the presence of different extracts of *Anemone baicalensis* aerial part; Figure S2: Evolution of absorbance of different extracts of *Anemone baicalensis* aerial part over time assessed by the nitric oxide scavenging method; Figure S3: Chemical structures of the compounds identified in the methanol extract of *Anemone baicalensis* aerial part; Figure S4: Positive-ion mode UHPLC-MS findings of methanol extract of *Anemone baicalensis* aerial part; Figure S5–S92: Presentation of MS and MS/MS spectra for peaks 1 through 44.
